# Optimized electroencephalogram and functional near-infrared spectroscopy-based mental workload detection method for practical applications

**DOI:** 10.1186/s12938-022-00980-1

**Published:** 2022-02-02

**Authors:** Hongzuo Chu, Yong Cao, Jin Jiang, Jiehong Yang, Mengyin Huang, Qijie Li, Changhua Jiang, Xuejun Jiao

**Affiliations:** 1 National Key Laboratory of Human Factors Engineering, China Astronaut Research and Training Center, Beijing, China; 2 Space Engineering University, Beijing, China

**Keywords:** EEG, fNIRS, Mental workload, Man–machine systems

## Abstract

**Background:**

Mental workload is a critical consideration in complex man–machine systems design. Among various mental workload detection techniques, multimodal detection techniques integrating electroencephalogram (EEG) and functional near-infrared spectroscopy (fNIRS) signals have attracted considerable attention. However, existing EEG–fNIRS-based mental workload detection methods have certain defects, such as complex signal acquisition channels and low detection accuracy, which restrict their practical application.

**Methods:**

The signal acquisition configuration was optimized by analyzing the feature importance in mental workload recognition model and a more accurate and convenient EEG–fNIRS-based mental workload detection method was constructed. A classical Multi-Task Attribute Battery (MATB) task was conducted with 20 participating volunteers. Subjective scale data, 64-channel EEG data, and two-channel fNIRS data were collected.

**Results:**

A higher number of EEG channels correspond to higher detection accuracy. However, there is no obvious improvement in accuracy once the number of EEG channels reaches 26, with a four-level mental workload detection accuracy of 76.25 ± 5.21%. Partial results of physiological analysis verify the results of previous studies, such as that the θ power of EEG and concentration of O_2_Hb in the prefrontal region increase while the concentration of HHb decreases with task difficulty. It was further observed, for the first time, that the energy of each band of EEG signals was significantly different in the occipital lobe region, and the power of $\beta_{1}$ and $\beta_{2}$ bands in the occipital region increased significantly with task difficulty. The changing range and the mean amplitude of O_2_Hb in high-difficulty tasks were significantly higher compared with those in low-difficulty tasks.

**Conclusions:**

The channel configuration of EEG–fNIRS-based mental workload detection was optimized to 26 EEG channels and two frontal fNIRS channels. A four-level mental workload detection accuracy of 76.25 ± 5.21% was obtained, which is higher than previously reported results. The proposed configuration can promote the application of mental workload detection technology in military, driving, and other complex human–computer interaction systems.

## Introduction

Mental workload has long been a factor of immense interest in designing and applying complex human–machine systems [1], and is increasingly recognized as a serious, worldwide public health concern. Only when the mental workload is in the appropriate range can high performance and operational reliability be maintained. An irregular mental workload state will impair a person’s work performance, leading to task failure [2, 3], and might endanger people’s health or safety in severe cases. In human–machine systems with high safety requirements, such as in the military, aviation, aerospace, driving, and other domains, human errors such as information acquisition and judgment decision error caused by excessive mental workload are often responsible for accidents [4, 5]. For example, soldiers in a battlefield must participate in warfare for a long time without interruption and must stay alert to respond to various systems. The harsh environment poses extraordinary physical and psychological challenges to them [6, 7]. Goodman et al. [8] reported that of 1094 USAF Unmanned Aerial Vehicle (UAV) operators stationed in the US, approximately 20% reported high fatigue, 11% reported high cynicism, and 3% reported reduced performance. This is because the long-term unsuitable mental workload state seriously endangers the operator’s mental and physiological health. Therefore, it is crucial to measure the mental workload of the operator accurately.

In the past several decades, neurophysiological signal have played an important role in mental workload detection because of its objectivity and stability[9]. One of the major topics to be investigated in this field is electroencephalogram (EEG)-based mental workload detection method. For instance, Georgios et al. [10] carried out a study of EEG based mental workload detection, and the experiment was implemented with N-back and mental arithmetic (the most commonly used single cognitive resource tasks for studies of mental workload [11–13]). The binary classification accuracies of mental workload are 88% and 86% in the N-back task and mental arithmetic task, respectively. Besides, there are still some researches on EEG for mental workload detection in complex tasks. Hongquan Qu et al. [14] carried out a three-level Multi-Task Attribute Battery (MATB)[15] task with 32-channel electroencephalogram (EEG) acquisition. Power spectrum density (PSD) was analyzed with independent components analysis (ICA) algorithm, and the average recognition accuracy reached 79.8%. 64-channel EEG data were recorded in a simulated flight experiment, and PSD, phase lag index (PLI) connection features were analyzed and extracted, giving a recognition accuracy 82% of three-level mental workload [16]. For application of mental workload detection, the significant advantage of EEG is that it contains abundant information, but it also has the disadvantages of low spatial resolution and complex operation.

As a new neurophysiological signal acquisition technology, recently, functional near-infrared spectroscopy (fNIRS) has become a research hotspot in this field with the advantages of high spatial resolution and portability [17]. Reported studies proposed that fNIRS performs well in mental workload detection of both single task and complex task. For example, Asgher et al. [18] observed the brain activities of the prefrontal cortex (PFC) region with fNIRS technology in a four-level mental arithmetic task, and obtained a recognition accuracy 89.31% with classification algorithm of long short-term memory (LSTM). Siddiquee et al. [19] explored the response difference of brain activity measured by fNIRS in different areas of the PFC. N-back task was conducted in the experiment, and the results show that the blood oxygen of middle prefrontal position can significantly improve the recognition precision, with a highest binary classification accuracy 90%. In a study of actual driving environment [20], four-channel fNIRS system was adopted to monitor the real-time change of blood oxygen in PFC region. The recognition accuracy of three-levels driving tasks reached 82.71%. Besides, in a study of air traffic control instructions tasks in flight simulators, Gateau et al. [21] collect fNIRS signals to detect the mental load state of pilots in two different group, and the accuracy reached 80% by SVM.

Aforementioned studies of mental workload detection are based on single physiological signal (either EEG, or fNIRS). Either the number of grades or the recognition accuracy of mental workload detection was not ideal; i.e., in Ref. [10] the authors reached an high accuracy but over a low number of classes, while in Ref. [16] the authors reported a classification performance on a high number of classes but with a low discrimination accuracy. This limitation is probably due to the limited information of single physiological signal. Therefore, researchers began to focus on multi-physiological signal fusion detection methods. Liu, Y. et al. [22] carried out a study on mental workload detection of simple cognitive resource tasks by fusing 28-channel EEG and 16-channel fNIRS. 3-level workload was induced by N-back task in the experiment, and the result indicates recognition accuracy based on fusion of EEG and fNIRS was significantly greater compared with single signal of EEG or fNIRS. Similar result was also observed in mental workload detection of complex tasks, Sangtae et al. [23] proposed a multi-physiological signal based mental workload detection method, which collected 64-channel EEG signals, and eight-channel fNIRS signals of drivers simultaneously. The result indicates that the recognition accuracy based on multi-physiological signal was significantly greater than that of single physiological signal. What’s more, reported literatures [11, 24] support the above viewpoint as well. In conclusion, existing studies suggest that the combination of multiple physiological signals can obtain better performance in mental workload detection compared with signal physiological signal. Nevertheless, there are still some limitations of the reported studies in the following aspects: the multiple physiological signals acquisition configuration was based on an excessive number of channels (i.e., 64 channels in the 10–20 system) [11, 23], only three or even less different levels of mental workload were considered [25–27], and the recognition accuracy was not sufficiently high (i.e., accuracy value higher than 70%).

In a related study to practical applications, Chi et al. [28] designed an experiment to collect task completion times and subjective mental load for five driving tasks. The results showed that task completion times for truck driving could be predicted using a learning curve. Practice significantly reduced mental workload ratings. Midha et al. [29] used fNIRS in an office environment to measure changes in mental workload for daily reading and writing tasks. In actual flight scenarios, Hankins et al. [30] collected ECG, EEG, EEG and subjective data to assess the level of mental load in different driving states. From these studies related to practical applications it is clear that the following issues must be paid attention to in real-time monitoring of mental workload in complex man–machine system. First, monitoring equipment should be portable and easy to operate, with a simple channel configuration of signal acquisition as far as possible. In addition, the response speed of the monitoring model should be as high as possible; consequently, algorithms with a large amount of computation are not suitable for this task. Finally, the gradation of the mental workload should be more detailed and the detection accuracy should be higher. This study focuses on these issues, and adopted complex simulation tasks to mimic the actual task scenarios. First, optimized data acquisition system integrates a few channels of EEG acquisition devices and a portable near-infrared devices was adopted. In addition, this study considers the modeling performance based on three different feature sets: derived from EEG signal, derived from fNIRS signal, derived from both EEG and fNIRS signals. Various classifiers were used to obtain an optimization modeling method. Last but not least, the difficulty of experiment task was divided into four levels, both of EEG and fNIRS features were took into account in the detection model of mental workload for a better modeling performance. The results of this study can promote the application of mental workload detection technology in military, driving, and other complex human–computer interaction systems.

## Results

### Behavioral data analysis results

**Fig. 1:**
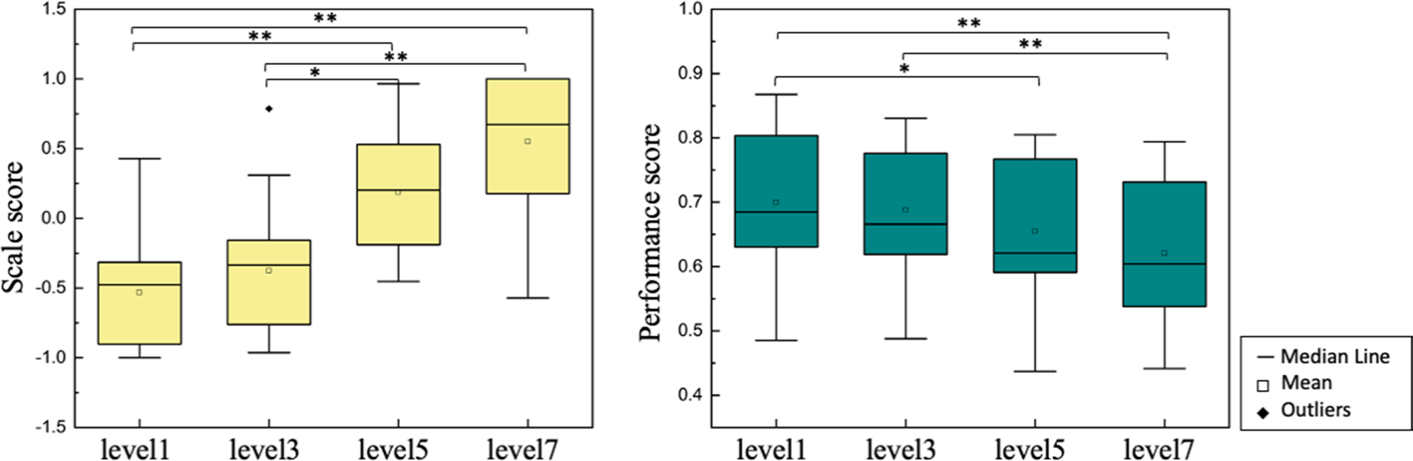
Subjective scale result and performance score result. The normalized subjective scale scores for each task difficulty are shown on the left, and the normalized performance scores for each difficulty are shown on the right. Note that the * means p_FDR < 0.05, ** means p_FDR < 0.01

Shown in Fig. 1, the influence of the task’s difficulty level on the comprehensive scale and comprehensive performance of 20 subjects was analyzed. Figure 1 indicates that subjective scale scores increase while the task performance decrease with the increase of task difficulty. After the outliers were removed, one-way ANOVA analysis showed that the overall score of the scale had a major effect on the mental workload level [F (3, 76) = 31.633, *P* < 0.001]. Post hoc analysis showed significant differences between load levels 1 and 5 (P_FDR < 0.01), 1 and 7 (P_FDR < 0.01), 3 and 5 (P_FDR < 0.05), and 3 and 7 (P_FDR < 0.01) (P_FDR < 0.05). One-way ANOVA analysis of performance scores showed that the main effect of combined performance scores was also observed on the mental workload level, F (3, 76) = 17.16, *P* < 0.001. Post hoc analysis showed that there were significant differences in the overall performance between difficulty 1 and 5 (p_FDR = 0.001), 1 and 7 (p_FDR < 0.001), and 3 and 7 (p_FDR = 0.001) (p_FDR < 0.05).

### Feature changes analysis results

#### EEG channels selection

**Fig. 2:**
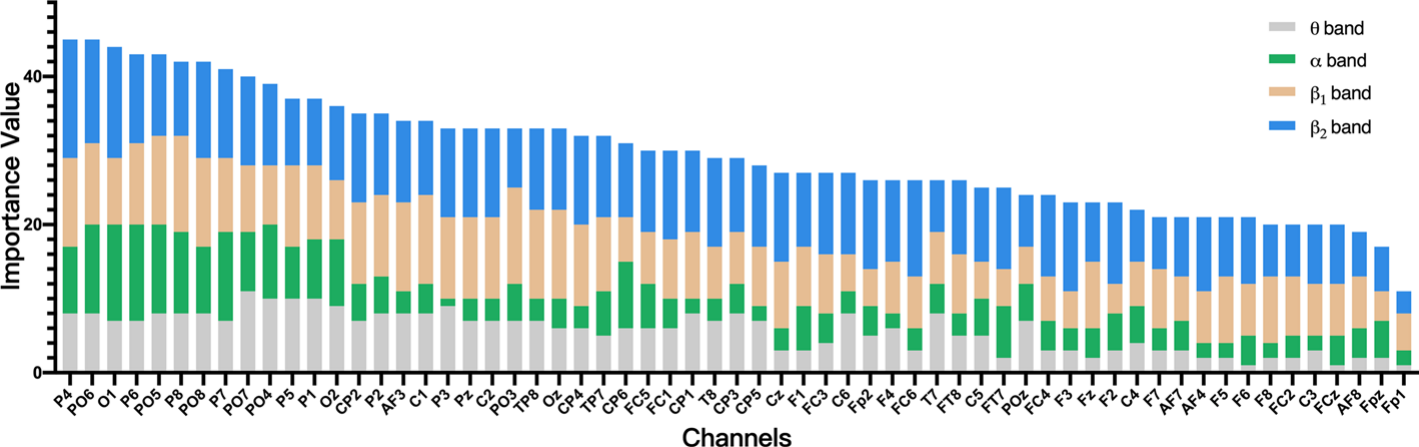
Channels importance ordering. The horizontal axis is channel, and the vertical axis is importance value of each channel, and the results were sorted from high to low. Different colors represent different frequency bands of EEG, and the height of the color block represents the features importance for a given channel

In order to obtain the optimal EEG channel configuration for measuring mental workload, the importance of each channel was calculated by the algorithm mentioned in 5.5.2 above, and the results were sorted from high to low, as shown in Fig. 2.

**Fig. 3:**
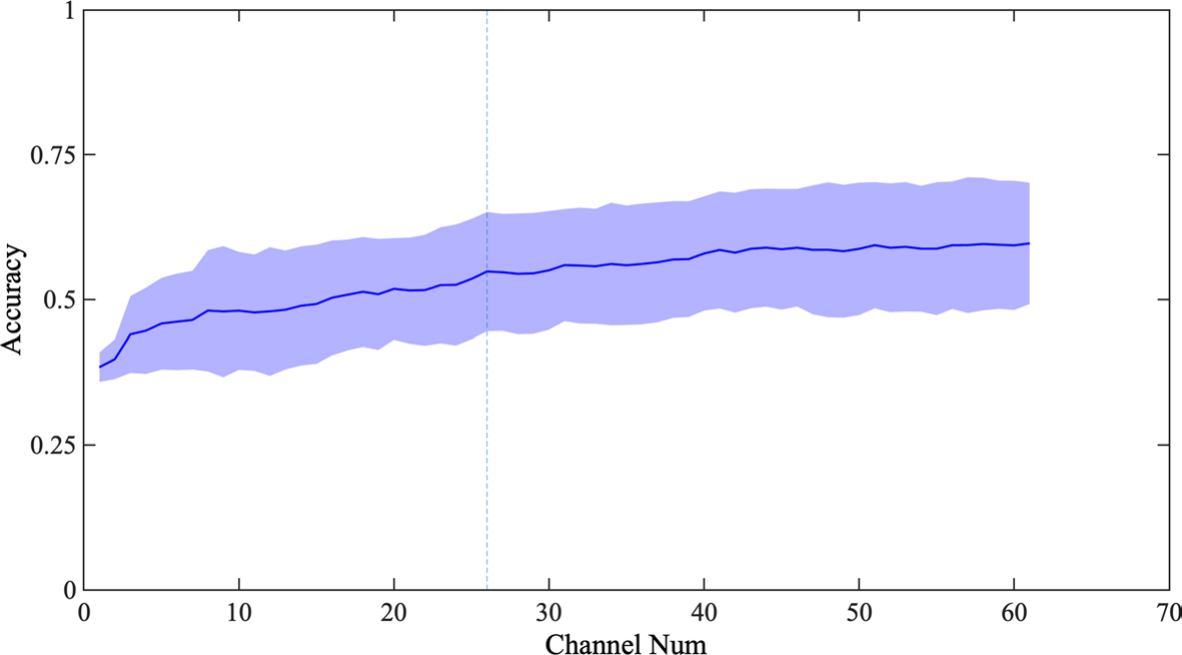
The accuracy varies with the number of channels. The figure shows the change curve of accuracy based on SVM under different number of channels. The horizontal axis is the number of channels and the vertical axis is the accuracy. The colored area is the variance of accuracy for different subjects, and the blue line represents the mean of the accuracy. The selection of channels is based on the sorting shown in Fig. 2

It can be seen in Fig. 3 that almost all channels have good performance in $\beta_{1}$ and $\beta_{2}$, which indicates that power of $\beta_{1}$ and $\beta_{2}$ are sensitive physiological characteristics of mental workload. In top-ranked channels (P4, PO6, O1, P6, PO5, P8, PO8, P7, PO7, PO4), the power of θ and α has a greater influence on the model. In addition, Fig. 3 reveals that the sensitive channels on mental workload of EEG mainly comprised occipital lobe (P4, PO6, O1, P6, PO5, P8, and OZ), the frontal (AF3) and sports area (C1, C2).

Furthermore, classification model based on SVM classifier was performed to explore the influence of channel number on classification accuracy. The channel was added to the model one by one based on the ranking order in Fig. 3, and the modeling performance varying with the number of channels is shown in Fig. 3. It can be seen that the classification accuracy of the model increases with the increase of the number of channels, but the rising trend slows down when the number of channels reaches 26. Therefore, we selected 26 channels (P4, PO6, O1, P6, PO5, P8, PO8, P7, PO7, PO4, P5, P1, O2, CP2, P2, AF3, C1, P3, Pz, C2, PO3, TP8, Oz, CP4, TP7, CP6) for subsequent research.

#### EEG feature changes analysis results

**Fig. 4:**
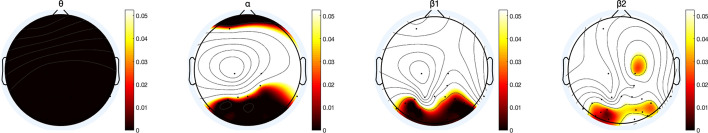
ANOVA of each channel comparing four difficulty levels. The figure shows ANOVA results in four frequency bands. All data are processed by Z-score first and outliers are removed. The result was corrected by FDR. White represents *P* > 0.05, deep brown indicates *P* < 0.01, and light brown indicates 0.01 < *P* < 0.05

ANOVA was performed to analyze the difference of power response in θ, α, $\beta_{1}$ and $\beta_{2}$ band under various mental workload levels. The statistical analysis results of 62 scalp electrodes was demonstrated as scalp maps in Fig. 4, and the result was corrected by FDR, since hundreds of comparisons were implemented simultaneously. After removing the outliers, ANOVA results showed differences in PSD that were mainly concentrated in all regions of θ band, prefrontal and occipital regions of α band, and occipital regions of $\beta_{1}$ and $\beta_{2}$ band.

**Fig. 5:**
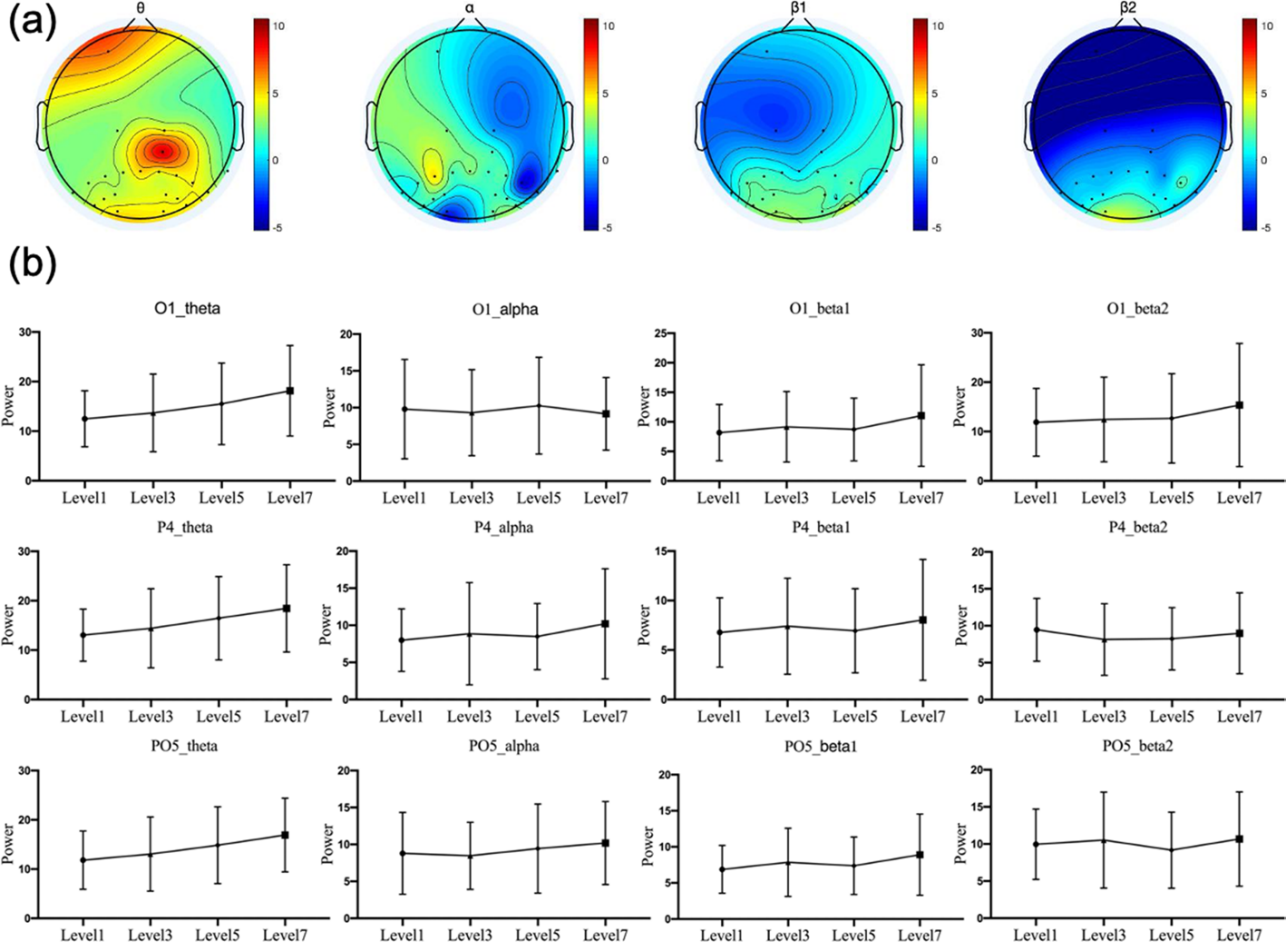
Power of different EEG band. **a** Shows the difference of power in *θ*, *α*, ${\beta }_{1}$ and ${\beta }_{2}$ band between highest load (Level 7) and lowest load (Level 1), and the result of 62 electrodes was averaged among 20 subjects in the scale map. **b** Shows the change trend of PSD in *θ*, *α*, β_1 and β_2 band of specific channel (O1, P4 and PO5) with task difficulty. The *x*-axis represents task difficulty level and the y-axis represents the power amplitude

Further, in order to find out the change trend of PSD with task difficulty, the difference of power in *θ*, *α*, $\beta_{1}$ and $\beta_{2}$ band between highest load (Level 7) and lowest load (Level 1) were calculated. The average results among 20 subjects of 62 scalp electrodes were demonstrated as scalp maps are shown as Fig. 5 a. Figure 5b indicates that power of θ band in prefrontal increased with the increase of task load, while power of α band in the right hemisphere and occipital region decreased with the increase of task difficulty. In addition, the power of $\beta_{1}$ and $\beta_{2}$ bands in the occipital region increased with the increase of task difficulty.

Specifically, the change trend of PSD in *θ*, *α*, $\beta_{1}$ and $\beta_{2}$ band of three typical channels (O1, P4 and PO5) were analyzed, as shown in Fig. 5a. According to the data shown in Fig. 5b, three major conclusions can be obtained: power of *θ* band in O1, P4 and PO5, showed a good correlation with task difficulty and increased with the increase of load level, which was consistent with the previous research [34]; power of *β*-band in O1 and PO5 also increased with the increase of task load; the *α*-band energy of O1 channel is negatively correlated with the load, which is consistent with the study [12].

#### fNIRS feature changes analysis results

**Fig. 6:**
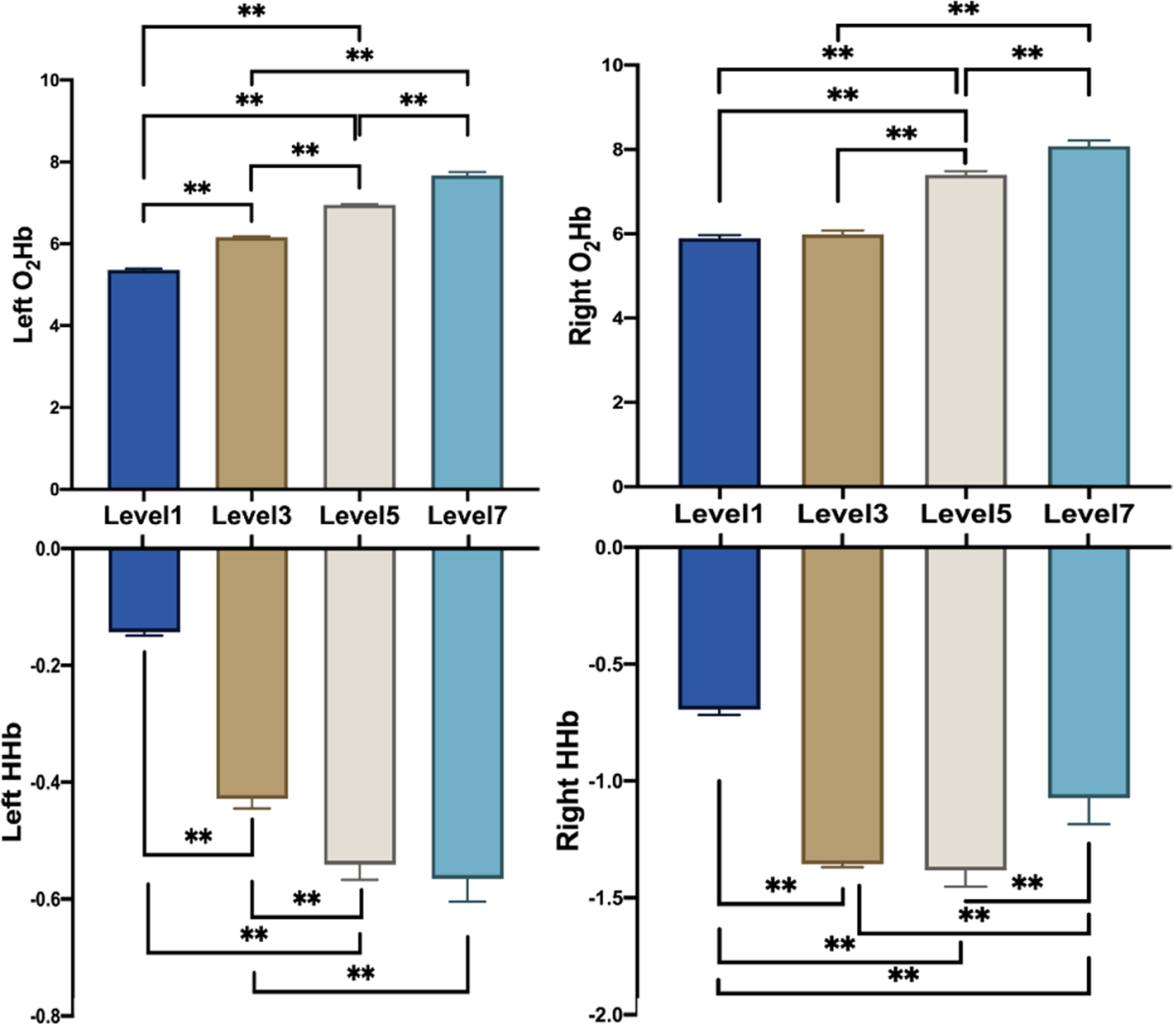
Changes of blood oxygen under different load levels. The left and right images represent the left and right sides of the forehead, with O_2_HB changes on the upper side and HHB changes on the lower side. Note that the * means p_FDR < 0.05, ** means p_FDR < 0.01

Next, the changes of O_2_Hb and HHb during the change of mental workload were analyzed. The average of 20 subjects under the same load level obtained the results as shown in Fig. 6, which shows the variation trend of O_2_Hb and HHb amplitude in the left and right prefrontal regions with a time window of 3 s. After the outliers were removed, one-way ANOVA analysis showed that the main effect of O_2_Hb on the left and right side was observed at the load level, *F* (3, 596) = 24,339.950, *P* < 0.001, *F* (3, 596) = 5499.275, *P* < 0.001. The main effect of HHb on the left and right side was also observed on the load level, *F* (3, 596) = 4455.428, *P* < 0.001, *F* (3, 596) = 2370.904, *P* < 0.001. Post hoc results showed significant differences among all grades (*P* < 0.01).

After the brain enters the working state, local neuronal activations increase metabolic rate, leading to increased blood flow and volume [36]. At the beginning of the task, PFC oxygenation locally increases, and the higher the load of the task, the more active the brain becomes, which also leads to the increase of O_2_Hb in the PFC region [37]. As can be seen from the pattern shown in the figure, during the period of Level 1 to Level 7 tasks, the concentration of O_2_Hb increases with the difficulty of tasks, and the content of O_2_Hb at Level 7 is significantly higher than that of other tasks with load levels. We also note that the change of O_2_Hb is more dramatic under high workload levels. Especially during a Level 7 task, the variation range and average amplitude of O_2_Hb are significantly improved compared with Level 1. The change of HHb and O_2_Hb showed an opposite trend. The content of HHb decreased with the difficulty of the task, and the change range of HHb was significantly lower than that of O_2_Hb.

### Classification results

In order to study the classification effect of EEG, fNIRS, and EEG–fNIRS feature sets, three classifiers, namely, SVM, RF, and DT, were performed to establish the recognition model. After processing, 960 samples per subject were obtained for each difficulty level, on a total of 3840 samples for the four difficulty levels. Only-EEG feature set is the dataset using only EEG features, and the dimensionality of the dataset is 3840 samples × 132 features. only-fNIRS feature set is the dataset using only fNIRS signal extraction features, and the dimensionality of the dataset is 3840 samples × 44 features. The EEG–fNIRS feature set uses both fNIRS features and EEG features, and the dimension of the dataset is 3840 samples × 180 features. All classification results were obtained using the 20% number of retained feature sets tested.

Table 1 presents the minimum, maximum, average and standard deviation value of classification accuracies of different feature sets among 20 subjects. For only-EEG feature set, the mean of classification accuracies of SVM, RF and DT are 52.29%, 54.24% and 45.89%, respectively, with a highest recognition accuracy 54.24% in RF classifiers. For only-fNIRS feature set, the mean of classification accuracies of SVM, RF, and DT are 67.13%, 70.30% and 62.163%, respectively, with a highest recognition accuracy 70.30% in RF classifiers. For EEG–fNIRS feature set, the mean of classification accuracies of SVM, RF, and DT are 70.23%, 76.25% and 64.46%, respectively, with a highest recognition accuracy 76.25% in RF classifiers.

**Table 1:** Test classification results on three feature sets

Feature set	Classifier	Results			
		Min.	Mean	Max.	Std.
Only-EEG	SVM	28.67	52.293	75.43	11.87
	RF	32.33	**54.245**	79.32	12.23
	DT	24.56	45.895	72.34	13.19
Only-fNIRS	SVM	52.20	67.132	82.10	8.43
	RF	51.92	**70.302**	81.23	8.27
	DT	50.89	62.163	77.21	7.85
EEG–fNIRS	SVM	63.39	70.233	83.71	6.34
	RF	69.46	**76.255**	88.20	5.21
	DT	53.35	64.464	80.38	7.33

Note that “Min” represents the minimum value of classification accuracy for all subjects, “Max” represents the Maximum value of classification accuracy for all subjects, “Mean” represents the average of the classification accuracy of all subjects, “Std” represents the standard deviation value in classification accuracy for all subjects

Bold represents the best results

To verify that our classifier is not overfitting, in Table 2, we give the results of the corresponding training set, and we can see that the test set results are very close to the training set results, and we can assume that our model is adequately fitted.

**Table 2:** Training classification results for the three feature sets

Feature set	Classifier	Results			
		Min.	Mean	Max.	Std.
Only-EEG	SVM	29.20	52.433	79.81	11.45
	RF	32.44	**55.473**	77.44	11.47
	DT	25.93	46.051	72.23	12.02
Only-fNIRS	SVM	54.27	67.722	83.13	8.40
	RF	52.98	**69.340**	80.88	7.48
	DT	50.55	62.390	76.62	7.58
EEG–fNIRS	SVM	62.00	73.853	85.08	6.29
	RF	70.49	**78.422**	88.51	4.15
	DT	54.90	67.473	81.00	7.79

Bold represents the best results

After removing outliers, a two-factor method was used to analyze the effects of various classifiers and datasets on the accuracy. The results showed that the main effect of accuracy was observed on the feature set and the classifier, *F* (2, 171) = 92.539, *P* < 0.01, *F* (2,171) = 15.253, *P* < 0.001. No interaction effect *F* (4, 171) = 0.317, *P* > 0.05 was observed. To sum up, RF classifier performed better in three feature sets compared with SVM and DT, and EEG–fNIRS feature set provided better performance than both only-EEG feature set and only-fNIRS feature set, with a highest four-level recognition accuracy 76.25 ± 5.21%.

What’s more, according to Table 1, we also observed that the standard deviations of recognition accuracy in EEG–fNIRS feature set was smaller than that of both only-EEG feature set and only-fNIRS feature set. In conclusion, EEG–fNIRS feature sets not only significantly improve the classification accuracy, but also make the model more stable and more robust, which is particularly important in practical applications.

## Discussion

In order to promote the practical application of the mental workload status detection technology, this study conducted MATB to simulate the cognitive needs of operators in their daily work and used portable EEG acquisition equipment and fNIRS acquisition equipment to collect the physiological signals of subjects during the task. In this study, first, EEG signals from 64 channels were simplified to 26 channels, which significantly improved the convenience of operating the equipment. Second, variation of both EEG and fNIRS features with the task difficulty were analyzed, in order to provide physiological explanation for the variation of mental workload. Finally, the modeling performance of only-EEG feature sets, only-fNIRS feature sets, and EEG–fNIRS feature sets in four levels of mental workload monitoring was explored and compared.

In the analysis of operator behavioral data, we observed that job performance significantly decreased and subjective scale score increase with the increase of mental workload. This indicates that excessive mental workload will lead to insufficient cognitive resources for operators to maintain good job performance. We selected 26 channels with the highest degree of correlation with load for analysis from the results of channel screening. It can be seen that these 26 channels are mainly concentrated in the occipital lobe and parietal lobe, while a few channels are located in the frontal region. The occipital lobe region is generally considered as a visual region [38], which is highly correlated with basic cognitive functions such as visual search and visual attention [39]. The performance of the operation is highly correlated with the parietal lobe [40]. The prefrontal region is involved in various higher cognitive abilities, such as executive function and memory [41]. This indicates that the mental workload of operators during the task is mainly caused by vision and operation, especially as the operator needs to take into account all subsystems, so it evokes a significant response in the visual region.

Further analysis of the changes in features found that the θ band power in the frontal region increased significantly with the increase of task load, and all brain regions showed significant differences. It can be considered that θ band power is a sensitive physiological index of mental load. When the mental load was increased, $\alpha$ band power in the occipital lobe and other hemispheric regions decreased, which was consistent with the results in reported studies [11, 42, 43]. In general, the power of $\beta_{1}$ and $\beta_{2}$ bands increased obviously with the increase of the load in the occipital region. However, by analyzing the power spectrum energy changes in specific channels, it was found that the power of $\beta_{2}$ band in O1 channel increased with the increase of task difficulty, while the power of $\beta_{2}$ band in P4 channel showed a downward trend. This shows that channel location has a substantial effect on features.

From the perspective of modeling performance, three different machine learning models were used to analyze the classification results under different feature sets. The results point out that the multimodal dataset is better than the unimodal dataset, which validates the results of [44–46]; moreover, the detection method established in this study is better in terms of classification accuracy than studies such as [11, 47] in the multimodal study. The comparison of the number of categories from load classification is higher than [18, 26, 48] and the robustness of the model is better. The reasons for the better results in this study are as follows: (1) the task selection, which is more relevant to the actual scenario, and the task difficulty setting is more reasonable, which effectively induces different levels of brain load; and (2) the channel screening was conducted before modeling, and some redundant EEG channels were eliminated before fusion with fNIRS features. This feature combination overcomes the defects of low EEG spatial resolution and low fNIRS temporal resolution on the one hand, and may generate other key interaction information to help improve the accuracy of EEG load recognition on the other hand. The feature data can be further mined later and relevant experiments can be designed to verify the present conjecture.

Finally, it should be noted that there are still some limitations in our study. For one thing, data acquisition configuration of EEG contains 26 electrodes, which means there is still an optimized space. For another, only an offline experiment was implemented in this study, and the experimental task is not a real application task scenario. For further exploration, we hope to reduce the number of EEG channels further and improve our measurement method by monitoring the mental workload status of pilots in real-time during flight missions. Furthermore, the absence of female subjects is a limitation and we will include female subjects in subsequent studies.

## Conclusions

In conclusion, this study was to construct a more accurate and convenient EEG–fNIRS-based mental workload detection method by optimizing the signal acquisition configuration. The result suggested 26 EEG channels and two frontal fNIRS channels is enough for a four-level mental workload detection accuracy of 76.25 ± 5.21%, which is higher than previously reported studies. The results of this study can promote the application of mental workload detection technology in military, driving, and other complex human–computer interaction systems.

## Method

### Participants

Twenty volunteers participated in our experiment, all from China Astronaut Research and Training Center, with an average age of 25.6 ± 2.24, normal or corrected to normal vision, without any neurological disease or history of neurological disease, and in a stable mental state before the experiment. In order to avoid the influence of gender differences and hand dominance on the results, all the subjects were male and right-handed. After familiarizing themselves with all the experimental procedures and requirements, the subjects signed the informed consent form.

During the experiment, the subjects were asked to sit in front of a 23-in. LCD monitor with their eyes about 70–90 cm away from the screen. The volunteers were asked to carefully read the instructions given on the screen before each sub-task and complete the experimental tasks as required. They were also asked to focus on the process of the experiment task execution as far as possible to get the best results, to ensure that the experimental data were real and effective, and to cooperate with data collection work.

### Experimental design

This experiment uses the MATB task to trigger the mental workload of subjects. This task is composed of three sub-tasks, namely, system monitoring, tracking, and resource management. It is a complex task involving three cognitive resources: attention, operation, and reasoning. The cognitive resources involved in the actual task are the same as those involved in the fundamental tasks performed by the operator, which can well simulate the real-world task environment of the subjects.

**Fig. 7:**
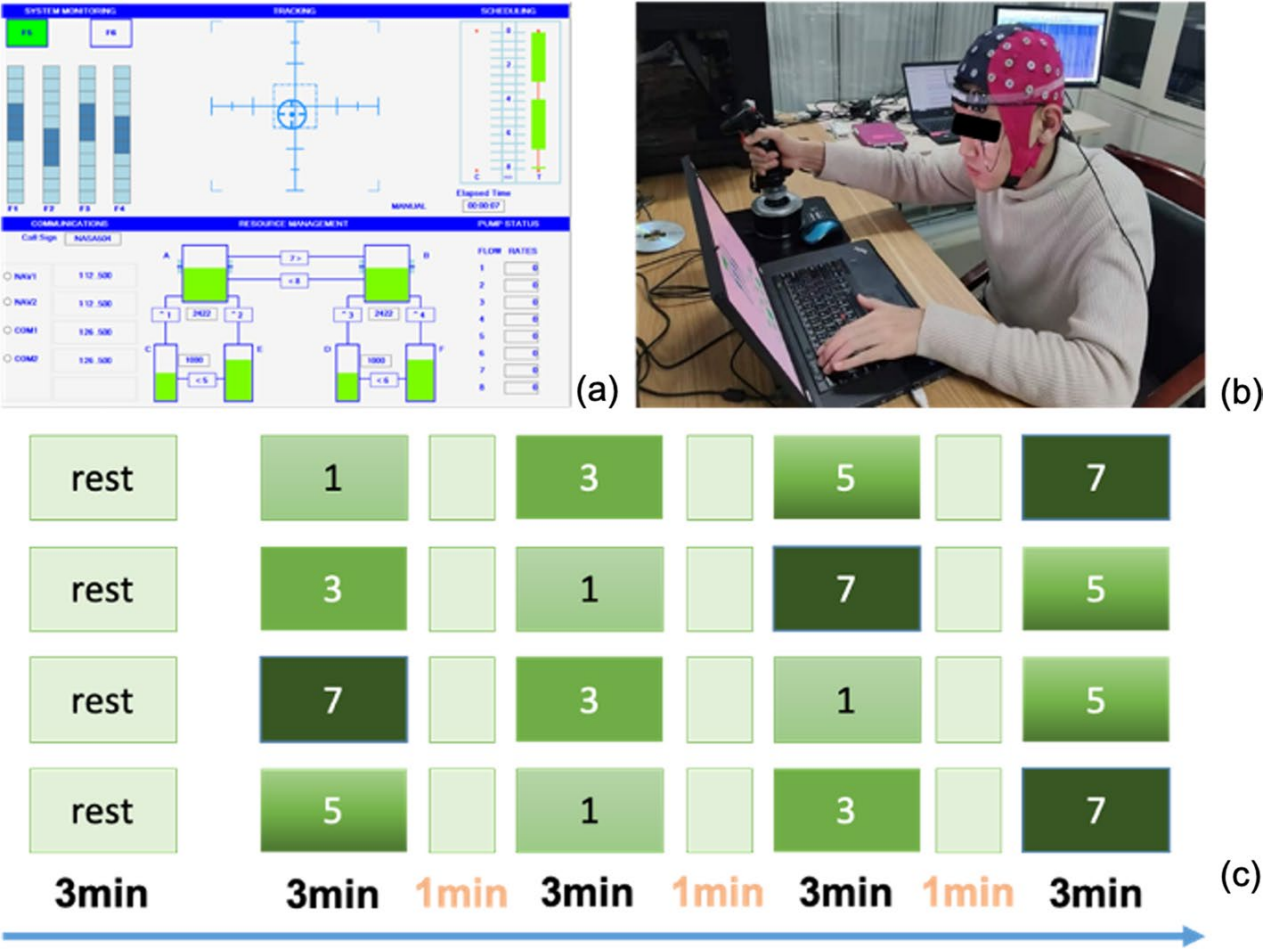
Stimuli and experimental procedure. **a** Shows the stimuli of the experiment paradigms. **b** Shows a real scenario of the experiment. **c** Demonstrates the experimental procedure. Note that the four different random stimuli sequence consisted of blocks repeated four times

During the task, three sub-tasks appear on the screen simultaneously. As shown in Fig. 7a, the upper left is the system monitoring task. When an abnormal state occurs, the subject will press the “F1–F6” button to respond, and the subject is required to press the corresponding button in the shortest possible time. On the upper right is the tracking task, in which subjects track a circular target by controlling a joystick and are asked to aim the crosshairs at the target as accurately as possible. The lower part is the oil management task. The subjects control the opening and closing of the oil circuit by pressing “1–8”, and they are required to ensure the oil volume of tank A/B remains within a specific range.

Experimental procedure is shown in Fig. 7c. The subjects completed a total of four blocks, each of which was randomly assigned four difficulty levels. Each block takes 3 min, and the four 3-min tasks are rated on a scale of difficulty from low to high: 1, 3, 5, and 7. There was a 1-min rest period between two tasks and a 3-min rest period before the task began. The actual experiment scene is shown in Fig. 7b. At the end of each task, the subjects were required to fill out the NASA-TLX scale. The four sequential tasks were performed in a random order among all subjects.

### Data acquisition

The EGGO device of Ant Neuro was used to record the EEG signals of the subjects at 64 electrode positions as per international standards 10–20. The reference was set at CPZ and the eye movement signals were recorded from below the left eye. The impedance of all electrodes is kept below 5 KΩ and the sampling rate is 500 Hz.

The PORTALITE device of Artinis was used to record the changes in blood oxygen in the brain on both sides of the forehead. The sampling rate is set to 50 Hz.

### Preprocessing and feature extraction

After the collected EEG data were obtained, the average values of the two mastoid channels (M1, M2) were used to re-reference the signals. Then, a 0.5–45-Hz Butterworth band-pass filter and a 50-Hz notch filter were conducted to remove the interference of the DC component, high-frequency component, and power frequency. The sampling rate was reduced to 200 Hz to reduce the data memory size. Independent component analysis (ICA) was adopted to remove eye movement, motion artifacts, channel noise, and other interference. Finally, the data were segmented every 3 s with the beginning of stimulus as the mark. The above processing uses the MATLAB open-source toolkit EEGLAB.

For each epoch, Welch’s method was used to extract the power spectral density (PSD), and the power of θ (3–8 Hz), α (8–13 Hz), β_1_ (13–20 Hz), and β_2_ (20–30 Hz) were obtained. Then, seven-channel pairs (P8–P7, O2–O1, C2–C1, P4–P3, PZ–O1, PZ–O2, and O1–AF3) were selected from the left and right brain as well as the front and rear brain, and the energy difference of each channel pair in the θ, α, β_1_, and β_2_ bands was calculated as the new feature. A total of 276 (62 channels × 4 bands + 7 channels × 4 bands) EEG features were extracted.

The collected fNIRS signal contains considerable noise, including motion artifacts, physiological interference, and instrument noise. Preprocessing was performed using the Homer2 open-source toolkit to remove the motion artifacts. A 0.5-Hz low-pass filter was also applied to reduce instrumental and physiological noise. The processing steps are consistent with Foy et al. [31, 32]. Finally, the data were segmented every 3 s, also marked by the time the stimulus started.

For each epoch, 11 statistical features were extracted, i.e., mean value, standard deviation, mean square error, skewness, root mean square, peak value, peak factor, kurtosis, waveform factor, pulse factor, and margin factor. A neurovascular coupling feature of the frontal EEG and frontal oxygen signals was added to calculate the zero-lagged correlation between the amplitude of HHb or O_2_Hb and the EEG frequency band power (in the four independent bands above). These HHb- or O_2_Hb-based NVO features are represented as oxidative neurovascular coupling and deoxy-neurovascular coupling, respectively. A total of 48 (2 channels × 2 × 11 + 2 channels × 2) fNIRS features were extracted.

To avoid disturbances from outliers, for EEG and fNIRS samples, we calculated the z-score of each subject’s samples to remove samples with greater than three times the standard deviation.

### Data analysis

#### Behavioral data analysis methods

Behavioral data collected in the course of this experiment mainly include the subjective scale score data filled out by the subjects and the task performance data generated during the task execution of the experimental platform. The subjects were asked to use the NASA-TLX scale to describe their subjective feelings of task load from six aspects: mental demand, physical demand, time demand, subjective evaluation of mission performance, effort, and frustration. To facilitate the analysis, the six dimensions of the subjective scale were normalized based on the maximum and minimum values and then averaged to obtain a comprehensive scale score. The MATB task used in this experiment consists of three sub-tasks: monitoring task, tracking task, and oil management task. Therefore, there are six performance indicators: the response time and response accuracy of the monitoring task; *X*- and *Y*-axis deviation of the tracking task; and fuel deviations of tank A and tank B for the oil management task. The performance data are normalized based on the maximum and minimum values and then averaged to obtain the comprehensive performance.

In order to analyze the influence of load level on tasks, we calculated Bonferroni-corrected ANOVA for overall performance as well as for comprehensive scale scoring, taking mental load level as a factor, and conducted post hoc comparison among various load levels. False discovery rate (FDR) correction was performed for multiple comparisons. The significance value was set at *P* < 0.05. By analyzing task behavior data, we can preliminarily summarize the law of the influence of task difficulty on mental load.

#### EEG channels selection methods

Given that not all areas of the human brain are directly related to mental workload, the 64-channel whole-brain EEG is unnecessary, and the fewer the channels, the easier it is to use in practice. The goal of this study was to use as few EEG channels as possible without losing accuracy. In order to find the brain regions that have high correlation with mental workload, the recursive feature elimination (RFE) algorithm based on SVM was used to filter the channels. RFE is a feature selection method with good performance and strong generalization ability [33]. The main idea is to select the best features by repeatedly building models (such as SVM), eliminating the selected features, and then repeating the above process on the remaining features until all the features are trawled. Considered to be physiological features closely related to mental load in reported studies [12, 34], PSD features of four frequency bands including *θ* (3–8 Hz), *α* (8–13 Hz), *β*_1_ (13–20 Hz), and *β*_2_ (20–30 Hz) of 64-channel EEG signals were calculated for screening. The top 100 features with the largest RFE results were selected of each subject for further analysis. Feature importance weight was defined as the number of people with this feature in the above-mentioned top 100 feature sets among 20 volunteers. Channel importance weight was defined as the sum of feature weights of four frequency bands of the channel.

#### Classification methods

In this study, three classification models, namely, Support Vector Machine (SVM), Decision Tree (DT), and Random Forest (RF), were adopted to model the extracted features. The grid search method was selected for different classifiers to obtain the optimal model parameters. Radial basis function (RBF) was applied as the kernel function in SVM, with two important parameters of C (punish coefficient) and error tolerance, with the search space as [0.0001, 0.001, 0.01, 0.1, 1, 10, 20, 30]. Parameter Gamma represents the number of support vector, with a search space of [0.1, 0.2, 0.25, 0.4, 0.8, 1.6, 3.2, 6.4]. The DT algorithm has three parameters that need to be adjusted: the partition standard, the maximum depth, and the minimum sample number required to segment the internal nodes. ‘Entropy’ and ‘Gini’, are tried as the search space of the parameter ‘partition standard’. The search space of parameter ‘maximum depth’ and parameter ‘Minimum sample number’ required to segment internal nodes is [10, 30, 60, 100] and [2, 5, 10, 15], respectively. RF is an ensemble classification model with good generalization. Reported studies [27, 35] have shown that Random Forest performs well in mental workload classification. The classifier mainly searches for the parameter “the number of Random Forest spanning trees”, with a adjusting space [100, 200, 500]. In addition, in order to ensure the reliability of the classification results, all classification algorithms are tested for final model performance by using 80% of datasets for a fivefold cross-validation and 20% number of datasets as a test set. The results section reports on the minimum, maximum, mean and variance of the classification results for all subjects. This will give a visual sense of the performance of the model.

## Data Availability

The datasets used and analyzed during the current study are available from the corresponding author on reasonable request.

## Abbreviations

EEG: Electroencephalogram
ICA: Independent component analysis
SVM: Support Vector Machine
MATB: Multi-task attribute battery
PSD: Power spectral density
PLI: Phase lag index
ERP: Event-related potential
DT: Decision Tree
RF: Random Forest
FDR: False discovery rate

## References

[1] Kuwato M, Hirano Y (2020). Sense of coherence, occupational stressors, and mental health among Japanese high school teachers in Nagasaki prefecture: a multiple regression analysis. BMC Public Health.

[2] Roy RN, Charbonnier S, Campagne A, Bonnet S (2016). Efficient mental workload estimation using task-independent EEG features. J Neural Eng.

[3] Rantanen EM, Goldberg JH (1999). The effect of mental workload on the visual field size and shape. Ergonomics.

[4] W. Chappelle, K. Mcdonald, and K. Mcmillan. Important and critical psychological attributes of USAF MQ-1 predator and MQ-9 reaper pilots according to subject matter experts. 2011.

[5] Mansikka H, Virtanen K, Harris D (2018). Dissociation between mental workload, performance, and task awareness in pilots of high performance aircraft. IEEE Trans Hum Mach Syst.

[6] El-Khodary B, Samara M (2018). The effect of exposure to war-traumatic events, stressful life events, and other variables on mental health of Palestinian children and adolescents in the 2012 Gaza War. Lancet.

[7] Iannacchione VG (2011). Validation of a research case definition of Gulf War Illness in the 1991 US Military Population. Neuroepidemiology.

[8] Chappelle W (2014). Assessment of occupational burnout in United States Air Force predator/reaper "drone" operators. Mil Psychol.

[9] Li R, Liu Z (2020). "Stress detection using deep neural networks. BMC Med Inform Decis Mak.

[10] Dimitrakopoulos GN (2017). Task-independent mental workload classification based upon common multiband EEG cortical connectivity. IEEE Trans Neural Syst Rehabil Eng.

[11] Liu Y, Ayaz H, Shewokis PA (2017). Multisubject “learning” for mental workload classification using concurrent EEG, fNIRS, and physiological measures. Front Hum Neurosci.

[12] Pergher V, Wittevrongel B, Tournoy J, Schoenmakers B, Van Hulle MM (2019). Mental workload of young and older adults gauged with ERPs and spectral power during N-Back task performance. Biol Psychol.

[13] Caywood MS, Roberts DM, Colombe JB, Greenwald HS, Weiland MZ (2017). Gaussian process regression for predictive but interpretable machine learning models: an example of predicting mental workload across tasks. Front Hum Neurosci.

[14] Qu H (2020). Mental workload classification method based on EEG independent component features. Appl Sci.

[15] Santiagoespada Y, Myer RR, Latorella KA, Comstock J. The multi-attribute task battery II (MATB-II) software for human performance and workload research: a user’s guide. Santiago-Espada, Yamira. 2011.

[16] Kakkos I (2019). Mental workload drives different reorganizations of functional cortical connectivity between 2D and 3D simulated flight experiments. IEEE Trans Neural Syst Rehabil Eng.

[17] Kohl SH, Mehler D, Lührs M, Thibault RT, Sorger B (2020). The potential of functional near-infrared spectroscopy-based neurofeedback—a systematic review and recommendations for best practice. Front Neurosci.

[18] Asgher U (2020). Enhanced accuracy for multiclass mental workload detection using long short-term memory for brain–computer interface. Front Neurosci.

[19] Siddiquee MR, Atri R, Marquez JS, Hasan S, Bai O (2020). Sensor location optimization of wireless wearable fNIRS system for cognitive workload monitoring using a data-driven approach for improved wearability. Sensors.

[20] Islam MR (2020). A novel mutual information based feature set for drivers’ mental workload evaluation using machine learning. Brain Sci.

[21] Gateau T, Durantin G, Lancelot F, Scannella S, Dehais F (2015). Real-time state estimation in a flight simulator using fNIRS. PLoS ONE.

[22] Liu Y, Ayaz H, Shewokis PA (2017). Mental workload classification with concurrent electroencephalography and functional near-infrared spectroscopy. Brain Comput Interfaces.

[23] Sangtae A, Thien N, Hyojung J, Kim JG, Jun SC (2016). Exploring neuro-physiological correlates of drivers’ mental fatigue caused by sleep deprivation using simultaneous EEG, ECG, and fNIRS data. Front Hum Neurosci.

[24] Cicalese PA (2020). An EEG-fNIRS hybridization technique in the four-class classification of Alzheimer’s disease. J Neurosci Methods.

[25] De A (2020). Prefrontal haemodynamics based classification of inter-individual working memory difference. Electron Lett.

[26] Le AS, Aoki H, Murase F, Ishida K (2018). A novel method for classifying driver mental workload under naturalistic conditions with information from near-infrared spectroscopy. Front Hum Neurosci.

[27] Pei Z, Wang H, Bezerianos A, Li J (2020). EEG-based multiclass workload identification using feature fusion and selection. IEEE Trans Instrum Meas.

[28] Chi CF, Cheng CC, Shih YC, Sun IS, Chang TC (2019). Learning rate and subjective mental workload in five truck driving tasks. Ergonomics.

[29] Midha S, Maior HA, Wilson ML, Sharples S (2021). Measuring mental workload variations in office work tasks using fNIRS. Int J Hum Comput Stud.

[30] Hankins TC, Wilson GF (1998). A comparison of heart rate, eye activity, EEG and subjective measures of pilot mental workload during flight. Aviat Space Environ Med.

[31] Foy HJ, Chapman P (2018). Mental workload is reflected in driver behaviour, physiology, eye movements and prefrontal cortex activation. Appl Ergon.

[32] Foy HJ, Patrick R, Peter C, Manabu S (2016). Prefrontal cortex activation and young driver behaviour: a fNIRS study. PLoS ONE.

[33] Qi C, Meng Z, Liu X, Jin Q, Su R (2018). Decision variants for the automatic determination of optimal feature subset in RF-RFE. Genes.

[34] Diaz-Piedra C, Sebastián MV, Di Stasi LL (2020). EEG theta power activity reflects workload among army combat drivers: an experimental study. Brain Sci.

[35] Novak D, Mihelj M, Munih M (2012). A survey of methods for data fusion and system adaptation using autonomic nervous system responses in physiological computing. Interact Comput.

[36] Tong Y, Lindsey KP, Hocke LM, Vitaliano G, Mintzopoulos D, Frederick B (2016). Perfusion information extracted from resting state functional magnetic resonance imaging. J Cereb Blood Flow Metab.

[37] Huppert TJ, Hoge RD, Diamond SG, Franceschini MA, Boas DA (2006). A temporal comparison of BOLD, ASL, and NIRS hemodynamic responses to motor stimuli in adult humans. Neuroimage.

[38] Liu D, Duan S, Wei P, Chen L, Zhang J (2020). Aberrant brain spontaneous activity and synchronization in type 2 diabetes mellitus patients: a resting-state functional MRI study. Front Aging Neurosci.

[39] Cui L (2019). Tai Chi Chuan vs general aerobic exercise in brain plasticity: a multimodal MRI study. Sci Rep.

[40] Ines RV (2017). Externally induced frontoparietal synchronization modulates network dynamics and enhances working memory performance. Elife.

[41] Xiao-Feng J, Liu J, Hua Q-F, Wu Y-J (2019). Relapsing-remitting multiple sclerosis is associated with regional brain activity deficits in motor- and cognitive-related brain areas. Front Neurol.

[42] Amihai I, Kozhevnikov M (2014). Arousal vs. relaxation: a comparison of the neurophysiological and cognitive correlates of vajrayana and theravada meditative practices. PLoS ONE.

[43] Rojas R (2020). Electroencephalographic workload indicators during teleoperation of an unmanned aerial vehicle shepherding a swarm of unmanned ground vehicles in contested environments. Front Neurosci.

[44] Sirpal P, Kassab A, Pouliot P, Dang KN, Lesage F (2019). fNIRS improves seizure detection in multimodal EEG-fNIRS recordings. J Biomed Opt.

[45] Ellen W, Jessica T, Nam CS, Franz JR (2017). Neuroimaging of human balance control: a systematic review. Front Hum Neurosci.

[46] Khan MU, Hasan M (2020). Hybrid EEG-fNIRS BCI fusion using multi-resolution singular value decomposition (MSVD). Front Hum Neurosci.

[47] Wilson GF, Russell CA (2003). Operator functional state classification using multiple psychophysiological features in an air traffic control task. Hum Factors.

[48] Aghajani H, Garbey M, Omurtag A (2017). Measuring mental workload with EEG+ fNIRS. Front Hum Neurosci.

